# Annotations

**Published:** 1903-12-05

**Authors:** 


### Annotations.

### Circulating Libraries for Poor-law Patients.

An interesting innovation has been made at the Portsmouth Parish Infirmary. For the use of the patients, and under certain restrictions, a circulating library has been established. The lady guardians were, we understand, the promoters of this scheme. They were struck by the enforced idleness of a number of people in the infirmary, and it seemed to them that some of their time might be properly and profitably employed by reading. If it had been proposed to purchase books for the use of the patients, objections might have been urged by the ratepayers, but the whole of the volumes which comprise the library have been the generous gifts of friends. The question of expense cannot therefore be raised. It is possible, however, that exception may be taken to the course pursued at Portsmouth on other grounds. Apprehensions may be felt lest the precedent should be applied in the case of workhouses. Certainly it would never do to start circulating libraries consisting of entertaining novels for the gratification of lazy, skulking men. But we do not know any reason why the hard lot of deserving paupers should not be rendered as bright as possible by persons who have the power and the will to mitigate it; and the idea of providing an assortment of books for the benefit of patients in the wards of the infirmary who are sufficiently well to peruse them, seems to us to be as unobjectionable in practice as it is kindly in intention. It is an indisputable fact that the advent of lady guardians has been followed by many excellent results in the treatment of inmates of workhouses; and we think that this last outcome of their sympathetic attitude to fellow-creatures less fortunate than themselves can only add to their well-merited popularity.

### Health Preservation among Weimar Scholars.

We once questioned a very bright little Italian boy as to what he was taught at school, and he gave a prominent place to "pretty behaviour." In their clumsy German way the educational authorities of Weimar appear to be aiming at something similar, partially disguised under a cloak of personal advantage, for under the heading "What We must Do to Keep Well" they have issued, and have caused to be suspended in all the primary schools of the Grand Duchy, a broadsheet containing the following 21 rules of conduct:—(1) We must wash our bodies, at least the face, neck, and chest, every day. (2) We must wash our hands often, and keep the nails short and clean. (3) We must clean our teeth with a brush every morning and after eating. (4) We must comb our hair morning and afternoon before going into school. (5) Our clothes must be daily cleansed from dust and dirt by beating and brushing. (6) Our shoes must be cleaned every morning. (7) We must cleanse them at the school door. (8) We must not throw fragments of paper, plants, food, or fruit upon the seats. (9) We must not spit on the floor. (10) In a warm room we must lay aside neck wraps and overcoats. (11) Fresh air must be admitted through the windows, especially between schoolhours. (12) We must spend the intervals as much as possible in the open air. (13) We must use the breakfast time for eating breakfast. (14) We must keep upright in walking, standing, and sitting. (15) In sitting we must keep both feet flat on the ground. (16) We must keep the body erect in reading, writing, and drawing. (17) We must write large and distinctly. (18) We must not write in our own shadows. (19) When at work, especially in reading, writing, and drawing, we must be screened from bright sunlight. (20) We must not read or write by twilight. (21) We must tell the teacher when it is either too hot or too cold in our place, when we cannot see or hear well from our place, when we feel ill, and when there is any illness in our homes. With a little editing, the Weimar broadsheet might not be without its uses in England, although it might possibly have a tendency to produce "passive resisters" among the scholars.

### Birth-rate and Death-rate in Argentina.

While North America is lamenting a falling-off in its native-born population and declaring that the increase of the United States is left to "the illiterate peasant women of Europe," South America seems to be increasing at a very satisfactory rate. This at least we infer from the "Annual Statistics of the Town of Buenos Ayres," a town where foreign influences, in which culture and sterility seem to go hand in hand, will be at least as powerful as anywhere else in South America. And there the native Argentine woman seems a reasonably prolific person, for at least one gave birth to her twenty-first child during last year. The Italian immigrant, however, does even better, for two of them reached the same number of offspring. Indeed, not only in such exceptional fertility, but all along the line, the Italian scores; the number of children born of Italian women during 1902 being 12,211, as against 8,411 of Argentine. As a whole, however, and whoever contributes to it, the birth rate of Buenos Ayres is a satisfactory one, the proportion being 37·2 per thousand inhabitants, while the rate in London is only 29·0. Indeed the Argentine capital stands lower only than three German towns, Cologne, Dusseldorf, and Nuremburg, and as its death rate is less than any of these, its general state may be regarded as rather better. Indeed, considering the notion that prevails among many people regarding the health of South American towns, it is worth while to emphasize the fact—all the more that business takes a considerable number of English there—that Buenos Ayres is a healthy town. Its death-rate is 16·2 per thousand inhabitants, while that of London is 18·0, of Glasgow 20·4, of Liverpool 22 8, and of Birmingham 18·9. The only large English town that can show a better record than Buenos Ayres is Leicester, the death-rate of which is only 15·1. It is satisfactory to our pride of race to find that the good condition of Buenos Ayres is due to the work and advice of a doctor with an Anglo Saxon surname, Don Guillermo Rawson, who predicted, in 1884, that if certain improvements were made, the death-rate of the town, which then stood at 30 per thousand, would fall to 18. Evidently the prediction has been more than verified, and that in spite of the fact that the population of the town is rapidly increasing, and that there are many immigrants who come from places where sanitation is little understood.

